# Comparison of high-flow nasal cannula oxygenation and non-invasive ventilation for postoperative pediatric cardiac surgery: a meta-analysis

**DOI:** 10.1186/s12890-024-02901-5

**Published:** 2024-02-21

**Authors:** Si-Jia Zhou, Xiu-Hua Chen, Ying-Ying Liu, Qiang Chen, Yi-Rong Zheng, Qi-Liang Zhang

**Affiliations:** Department of Cardiac Surgery, Fujian Children’s Hospital (Fujian Branch of Shanghai Children’s Medical Center), College of Clinical Medicine for Obstetrics & Gynecology and Pediatrics, Fujian Medical University, Fuzhou, China

**Keywords:** HFNC, NIV, Pediatric patients, Congenital heart surgery, Meta

## Abstract

**Objective:**

To evaluate the efficacy of high-flow nasal cannula oxygenation (HFNC) versus non-invasive ventilation (NIV) in pediatric patients post-congenital heart surgery (CHS) through a meta-analysis.

**Methods:**

A comprehensive literature search was conducted across the Chinese biomedical literature database, Vip database, CNKI, Wanfang, PubMed, Embase, Cochrane Library, and Web of Science until December 20, 2022. We selected RCTs or cohort studies that met inclusion criteria for a meta-analysis using RevMan 5.4 software.

**Results:**

Our search yielded five publications, comprised of one randomized controlled trial and four cohort studies. Meta-analysis revealed a significant reduction in reintubation rates in children post-CHS treated with HFNC as compared to NIV [RR = 0.36, 95%CI(0.25 ~ 0.53), *P* < 0.00001]. There was also a notable reduction in the duration of ICU stay [MD = -4.75, 95%CI (-9.38 ~ -0.12), *P* = 0.04]. No statistically significant differences were observed between HFNC and NIV in terms of duration of mechanical ventilation, 24 h PaO_2_, and PaCO_2_ post-treatment (P > 0.05). Furthermore, both groups showed no significant difference in the duration of extracorporeal circulation [MD = -8.27, 95%CI(-17.16 ~ 0.62), *P* = 0.07].

**Conclusions:**

For pediatric patients post-CHS, HFNC appears to be more effective than NIV in reducing reintubation rates and shortening the CICU stay.

## Introduction

With the advancements in medical technology, surgical intervention for congenital heart disease (CHD) in pediatric patients is being initiated at increasingly younger ages and at lower weights. Given the high rate of postoperative complications and rapid disease progression in pediatric CHD patients, particularly the elevated incidence of hypoxemia following extracorporeal circulation, precise and prompt respiratory management is paramount [1]. Traditional non-invasive ventilation approaches encompass conventional oxygen therapy (COT) and non-invasive mechanical ventilation (NIV). However, these modalities often fail to deliver the requisite oxygen concentration and flow post-extubation. Additionally, the inhalation of inadequately humidified, dry, and cold gas can result in complications such as nasal mucosal dryness and bleeding, compromising patient tolerance [2].

Recently, high-flow nasal cannula oxygenation (HFNC) has emerged as a promising solution, delivering a high flow (8–80 L/min) of gas at a regulated oxygen concentration (21%-100%), temperature (31–37 °C), and humidity via a high-flow nasal conduit [3]. As a novel respiratory support technique, HFNC has gained significant traction in clinical settings and is versatile across diverse age groups, from neonates and children to adults [4].

Aiming Liu et al. showed that HFNC has a good therapeutic effect in patients with AECOPD and type II respi-ratory failure. It improves the blood gas parameters, patient comfort and has clinical value [5]. HFNC has become a go-to solution for managing acute hypoxic respiratory failure in children and in averting reintubation post-extubation. While HFNC is frequently employed in pediatric intensive care units (ICU), there's a dearth of prospective research on its efficacy in post-extubation hypoxemia following pediatric cardiac procedures, with varying conclusions. Meta-analyses focusing on HFNC's efficacy post-pediatric cardiac surgery are limited, though more comprehensive investigations exist for adult post-cardiac surgery contexts [6–9]. Consequently, the quest for the optimal non-invasive respiratory support technique is ongoing, aiming to mitigate morbidity and mortality while maximizing the success of weaning off mechanical ventilation in pediatric post-CHS patients. This study endeavors to contrast the therapeutic outcomes of HFNC against NIV post-mechanical ventilation in CHS patients, with the goal of pinpointing a more effective and clinically feasible intervention.

## Information and methods

This meta-analysis were performed in accordance with the PRISMA guidelines and registered on PROSPERO.

### Literature search strategy

Our search combined both MeSH (Medical Subject Headings) terms and free-text terms to comprehensively query databases including the Chinese biomedical literature database, Vip database, CNKI, Wanfang, PubMed, Embase, Cochrane Library, and Web of Science. Searches were conducted in both English and Chinese from each database's inception to December 2022. We also manually searched references and related articles from the included literature for further pertinent studies. Using PubMed as an example, the combined search strategy is articulated as follows: (("Cannula"[Mesh]) OR (((((Cannulae[Title/Abstract]) OR (Nasal Cannula[Title/Abstract])) OR (Cannula, Nasal[Title/Abstract])) OR (Nasal Cannulae[Title/Abstract])) OR (Cannulae, Nasal[Title/Abstract]))) AND (("Cardiac Surgical Procedures"[Mesh]) OR ((((((((((((Procedure, Cardiac Surgical[Title/Abstract]) OR (Procedures, Cardiac Surgical[Title/Abstract])) OR (Surgical Procedure, Cardiac[Title/Abstract])) OR (Surgical Procedures, Cardiac[Title/Abstract])) OR (Surgical Procedures, Heart[Title/Abstract]))) OR (Cardiac Surgical Procedure[Title/Abstract])) OR (Heart Surgical Procedures[Title/Abstract])) OR (Procedure, Heart Surgical[Title/Abstract])) OR (Procedures, Heart Surgical[Title/Abstract])) OR (Surgical Procedure, Heart[Title/Abstract])) OR (Heart Surgical Procedure[Title/Abstract]))). Additional search keywords included: "high-flow oxygen therapy," "high-flow nasal cannula," "nasal high-flow oxygen therapy," "HFNC," "HHFNC," "HHFN," "Congenital Heart Disease," "Heart Abnormality," "Malformation of Hearts," "infant," "child," and "pediatrics," among others.

### Literature inclusion and exclusion criteria

Literature inclusion criteria: (1) study type: randomized controlled trial (RCT) or cohort study; (2) study population: withdrawal from mechanical ventilation in children after CHS; (3) management measures: treatment with HFNC and NIV. Literature exclusion criteria: (1) duplicate publications; (2) animal trials, conference articles, academic articles; (3) literature for which the full text was not available or access to the data had failed; (4) literature for which the statistical indicators required for this study were not available after reading the complete text, or for which statistical indicators were available but data extraction was not possible, and attempts to contact the authors had failed.

### Literature selection and data extraction

Two researchers independently screened the articles by reviewing the titles and abstracts based on the inclusion and exclusion criteria. Articles that did not meet the criteria were excluded. For the potentially relevant articles, a full-text review was conducted to finalize the selection. In cases of disagreement, the issues were discussed and resolved, or a decision was made by a third researcher. The extracted literature covered: (1) general information: including authors, year of publication, and sample size; (2) characteristics of study subjects: including gender and age; (3) interventions: including treatment and control measures; (4) outcome indicators: ① reintubation rate ② ICU stay duration ③ extracorporeal circulation duration ④ mechanical ventilation duration ⑤ PaO_2_ at 24 h after application of oxygen therapy ⑥ PaCO_2_ at 24 h after application of oxygen therapy; (5) type of study.

### Risk of bias assessment in the literature

To evaluate the methodological quality of the RCTs, we utilized the guidelines from the Cochrane Handbook for Systematic Reviews of Interventions [10]. Two researchers independently assessed the RCTs for potential bias using the risk of bias assessment tool recommended by the Cochrane Handbook. For cohort studies, methodological quality was appraised using the Newcastle–Ottawa Scale (NOS), with a maximum score of 9. In cases of disagreement, the discrepancies were discussed and resolved, or adjudicated by a third researcher.

The RCT evaluation comprised seven domains: random sequence generation, allocation concealment, blinding of participants and personnel, blinding of outcome assessment, incomplete outcome data, selective reporting, and other potential sources of bias. Each domain was categorized as having a low, unclear, or high risk of bias based on the assessment criteria. These risks were designated as “L” for low risk of bias, “U” for unclear risk, and “H” for high risk. Based on these categorizations, the overall quality of the studies was ranked as “A” if they fully met the criteria, “B” if they partially met them, and “C” if they did not comply at all.

The NOS criteria for cohort studies included three main sections and eight domains, totaling a possible score of 9. Scores less than five were deemed low quality, scores between 5 and 7 were considered moderate quality, and scores between 8 and 9 were categorized as high quality.

### Statistical analysis

Data processing and graphical representations (forest plots) were conducted using RevMan 5.4 software. For continuous variables, the effect measure was presented as the weighted mean difference (MD) with a 95% confidence interval (CI). For dichotomous variables, the risk ratio (RR) was utilized. Study heterogeneity was assessed using the X^2^ test. If P ≥ 0.05 and I^2^ < 50%, the studies were deemed to have low heterogeneity, and a fixed-effects model was employed for the analysis. Conversely, if *P* < 0.05 and I^2^ ≥ 50%, the studies demonstrated significant heterogeneity, prompting the use of a random-effects model. In cases of identified heterogeneity, we probed its source, conducted subgroup analyses to pinpoint the root of the heterogeneity, or executed a sensitivity analysis. Funnel plots were employed to assess potential publication bias among the studies. The significance level for the meta-analysis was set at α = 0.05, with P-values less than 0.05 indicating statistical significance.

## Results

### Results of the literature search

**Fig. 1:**
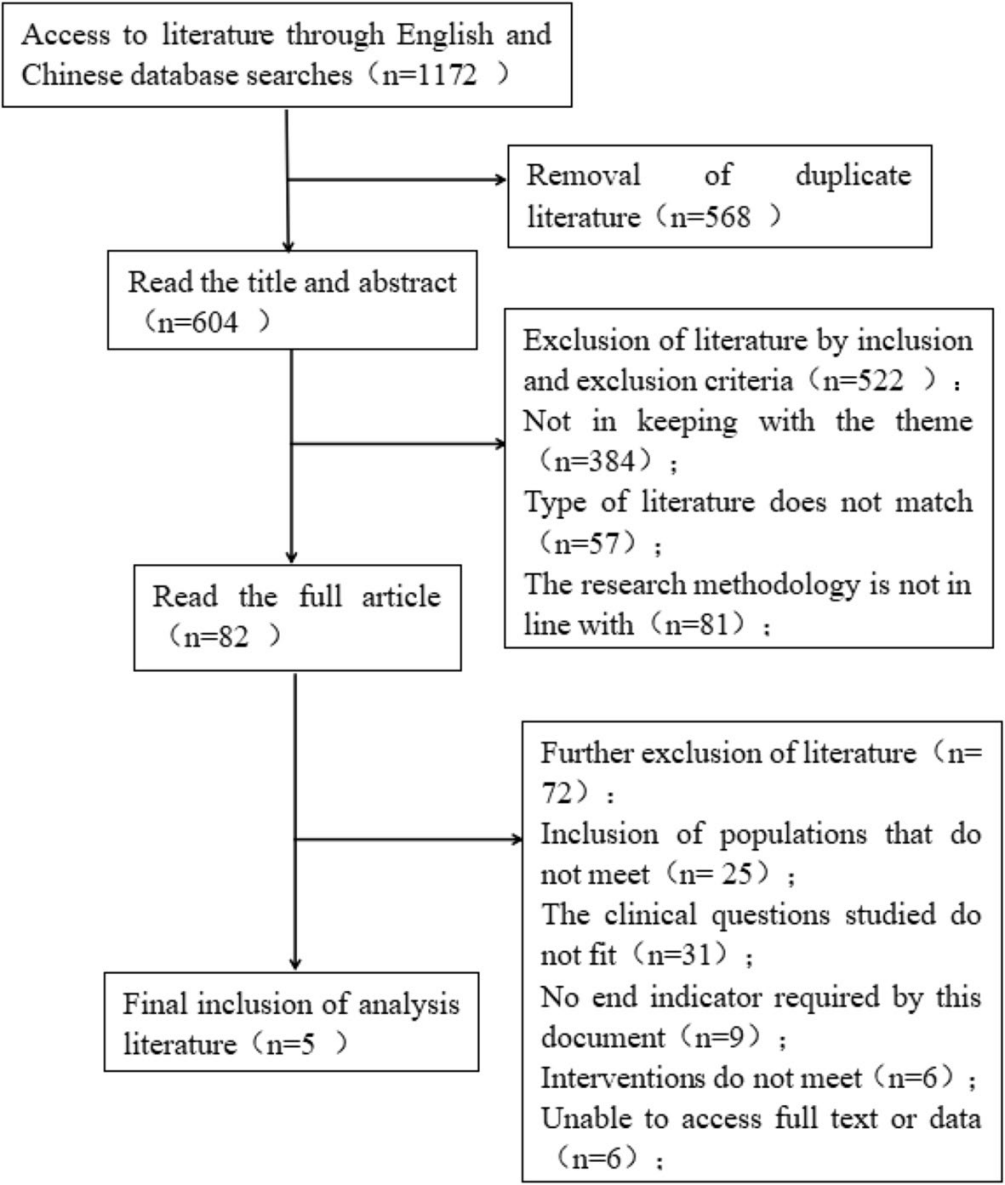
Flow chart of literature screening

In the preliminary literature search, we identified 1,172 articles. Ultimately, five studies were included: one RCT and four cohort studies. These studies encompassed a total of 813 pediatric patients, with 515 in the HFNC group and 298 in the NIV group. The process and results of literature screening are depicted in Fig. 1, while the key characteristics of the selected studies are detailed in Table 1. Detailed information about the included studies are shown in Table 2.

**Table 1:** Basic characteristics of the five included articles

Author	Year of publication	Sample size (cases)		Gender(example) [M/F]		Age		Interventions		Ending indicators	Type of research
		Observation group	Control group	Observation group	Control group	Observation group	Control group	Observation group	Control group		
Alok	2022	61	60	32/29	34/26	46.8 ± 36.3 m	43.2 ± 32.3 m	HENC	NIV	①②③④⑤⑥	RCT
Asaad G	2022	320	104	175/145	43/61	115. 0 ± 86.5d	26.0 ± 58.2d	HFNC	NIPPV	①②③④	Cohort study
Jessin	2020	50	50	25/25	26/24	6.94 ± 4.04 m	2.68 ± 2.97 m	HFNC	N/BiPAP	①③⑤⑥	Cohort study
Naohiro	2019	35	35	19/16	21/14	3.0 ± 1.6 m	1.0 ± 0.8 m	HFNC	NIV	①②④⑥	Cohort study
Robert P	2018	49	49	33/16	31/18	13.0 ± 65.3d	24.0 ± 57.4d	HFNC	PAP	①②③④	Cohort study

①Reintubation rate ② ICU length of stay ③ Extracorporeal circulation time ④ Mechanical ventilation time ⑤ 24 h PaO_2_ ⑥ 24 h PaCO_2_

**Table 2:** Summary of study findings

	Primary outcomes	Other outcomes	Types of surgery	Complications (HFNC:NIV)	STAT score (HFNC/NIV)
Alok	CO_2_ clearance pO_2_ pO_2_/FiO_2_	CPB,AXC,Respiratory rate,Heart rate, Mean blood pressure,Pulse oximetry, MV duration,ICU stay,Duration of oxygen ther apypost,Extubation, Postoperative transfusions, Complication rates	Ventricular septal defect 72 Aseptal defect 42 Pulmonary stenosis 7	Airway manipulation/use of airway adjuncts 4:6 Need to increase oxygen flow rate 3:2 Desaturation event 1:1 Hypotension 15:17 Bradycardia 1:0 Abdominal distension 4:7 Reintubation rate 1:2	NR
Asaad G	Extubation failure Etiology of extubation failure	Chromosomal abnormality,Genetic syndrome,Antenatal diagnosis,Single ventricle,STAT score,Fluid overload, Duration of MV,CPB Time,Cross-clamp time,Postop LOS,CICU LOS, Hospital LOS,Operative mortality	NR	NR	1 score 91:16 2 score 103:23 3 score 32:12 4 score 73:44 5 score 18:8
Jessin	Reintubation Complications	CPB time,ACC time, Surgical procedure, Mean PO_2_ at 24 h postextubation, Mean PCO_2_ at 24 h postextubation, Ventilation hours	Ventricular septal defect repair 29 Tetralogy of Fallot repair 23 Arterial switch operation 11 TAPVC repair 7 Glenn shunt 6 Atrioventricular septal defect repair 5 PDA ligation 5 Coarctation of aorta repair 4 Pulmonary artery banding 3 Systemic to pulmonary artery shunt 2 ALCAPA repair 2 Miscellaneous 3	Pneumothorax 1:1 Abdominal distension 0:8 Pressure ulcers related to device-interface 7:43	NR
Naohiro	Reintubation	MV time,Palliative surgery,CPB%,CPB time,Operative time,Blood loss, Duration of HFNC,Duration of NIV, Time to diagnosis of ARF after extubation,PaCO_2_,SaO_2_,SBP,RR,HR, ICU LOS	NR	NR	NR
Robert P	Extubation failure Hospital resource utilization	Genetic or congenital anomaly,Airway anomaly,Intubated prior to surgery, Single ventricle physiology,STAT category,CBP time,Mortality, Respiratory support time, Postsurgical hospital LOS	NR	NR	1–3 csore 29:30 4–5 score 20:19

*NR* No records, *CPB* Cardiopulmonary bypass, *AXC* Aortic cross-clamp time, MV Mechanical ventilation, LOS Length of Stay, ACC Aortic cross clamp, SBP Systolic blood pressure, RR Respiratory rate, HR Heart rate

### Quality assessment of included studies

**Fig. 2:**
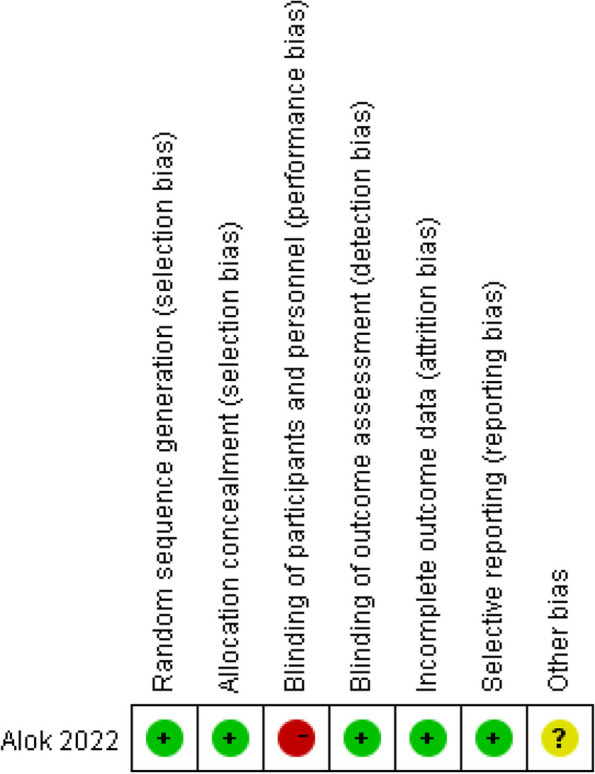
Assessment of risk bias

The five selected studies were well-matched at baseline. The methodological quality of the single RCT was evaluated using the Cochrane's risk of bias assessment tool. Due to the specific nature of the intervention (distinctive oxygen therapies), it was challenging to ensure complete blinding for the researchers. As a result, there was a recognized high risk of bias in the blinding domain, leading to a grade of 'B' for the study quality. This is visualized in Fig. 2. The four cohort studies were assessed using the Newcastle–Ottawa scale: three were deemed high-quality and one was of moderate quality, as shown in Table 3.

**Table 3:** NOS quality evaluation score table

Cohort stude	Selection of research subject	Comparability between groups	Ending comment	TTotal NOS score
Asaad G 2022	4	2	2	8
Jessin 2020	4	2	1	7
Naohiro 2019	4	2	2	8
Robert P 2018	4	2	2	8

### Meta-analysis results

#### Comparison of reintubation rates between HFNC and NIV

**Fig. 3:**
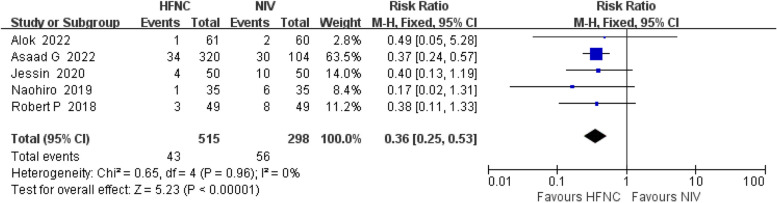
Forest plot of reintubation rates

Five studies [11–15] examined the effects of HFNC versus NIV on reintubation rates in post-CHS pediatric patients. There was no significant heterogeneity among these studies (*P* = 0.96, I^2^ = 0%). Using a fixed-effects model, there was a significant reduction in the reintubation rates with HFNC compared to NIV [RR = 0.36, 95%CI(0.25—0.53), *P* < 0.00001]. The results are presented in Fig. 3.

#### Comparison of ICU stay duration between HFNC and NIV

**Fig. 4:**
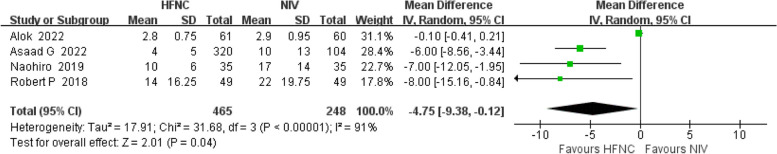
Forest plot of ICU length of stay

Four studies [11, 12, 14, 15] analyzed the impact of HFNC versus NIV on ICU stay duration in post-CHS pediatric patients. With noticeable heterogeneity present (*P* < 0.05, I^2^ = 91%), a random-effects model was employed. There was a significant reduction in ICU stay duration for the HFNC group as compared to the NIV group [MD = -4.75, 95%CI(-9.38—-0.12), *P* = 0.04]. These findings are illustrated in Fig. 4.

#### Comparison of extracorporeal circulation duration between HFNC and NIV

**Fig. 5:**
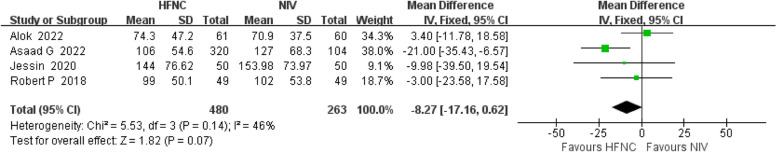
Forest plot of the duration of extracorporeal circulation

Four studies [11–13, 15] discussed the duration of extracorporeal circulation in pediatric CHS patients. Given the lack of significant heterogeneity (*P* = 0.14, I^2^ = 46%), a fixed-effects model was used. There was no significant difference between the HFNC and NIV groups concerning the duration of extracorporeal circulation [MD = -8.27, 95%CI(-17.16—0.62), *P* = 0.07]. The results are depicted in Fig. 5.

#### Comparison of mechanical ventilation duration between HFNC and NIV

**Fig. 6:**
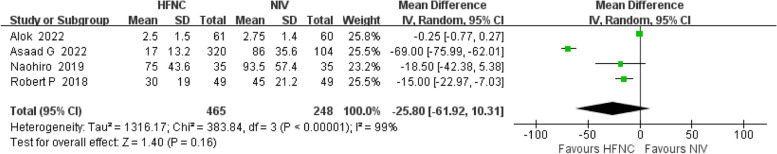
Forest plot of the duration of mechanical ventilation

Four studies [11, 12, 14, 15] investigated the effects of either HFNC or NIV on the duration of mechanical ventilation in post-CHS pediatric patients. Significant heterogeneity was observed (*P* < 0.05, I^2^ = 99%), so a random-effects model was applied. No significant difference was found between the HFNC and NIV groups concerning the duration of mechanical ventilation [MD = -25.80, 95%CI(-61.92—10.31), *P* = 0.16]. These findings are presented in Fig. 6.

#### Comparison of 24 h PaO_2_ and PaCO_2_ following oxygen therapy between HFNC and NIV

**Fig. 7:**
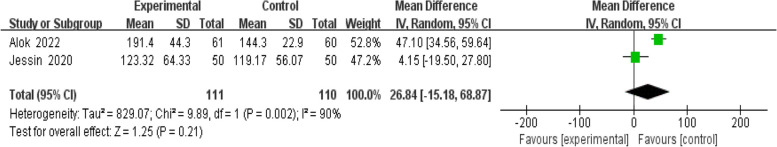
Forest plot of 24 h PaO_2_ after oxygen therapy

**Fig. 8:**
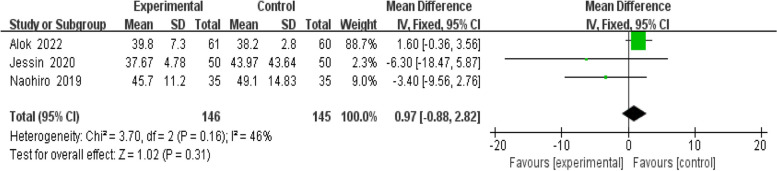
Forest plot of 24 h PaCO_2_ after oxygen therapy

Two studies [11, 13] analyzed PaO_2_ levels 24 h after the application of HFNC or NIV. Due to substantial heterogeneity (*P* < 0.05, I^2^ = 90%), a random-effects model showed no significant difference in 24 h PaO_2_ levels between the HFNC and NIV groups [MD = 26.84, 95%CI(-15.18—68.87), *P* = 0.21]. The results are detailed in Fig. 7. Three studies [11, 13, 14] evaluated the influence of HFNC versus NIV on 24 h post-oxygen therapy PaCO_2_ levels in post-CHS pediatric patients. Given the homogeneity (P > 0.05, I^2^ = 46%), a fixed-effects model was used. There was no significant difference in 24 h PaCO_2_ levels between the HFNC and NIV groups [MD = 0.97, 95%CI(-0.88—2.82), *P* = 0.31]. The findings are shown in Fig. 8.

### Sensitivity analysis

Using RevMan 5.4 software, effect sizes were combined for each outcome. Each included study was then sequentially excluded, and effect sizes recalculated. The heterogeneity among studies remained consistent, and the direction of meta-analysis results remained largely unchanged, suggesting the robustness of our findings.

## Discussion

Congenital heart disease (CHD) is prevalent in children and often necessitates surgical intervention to enhance survival and quality of life. Due to the underdeveloped state of children's organs, pediatric cardiac surgery (CHS) poses specific risks, leading to varying degrees of postoperative complications. Post-CHS, children can experience respiratory failure due to fluid retention, heightened pulmonary vascular resistance, muscle fatigue, diaphragmatic weakness, and edema [16]. This exacerbates medical challenges and directly influences patient outcomes. Proactive postoperative respiratory management, including the judicious selection of oxygen therapy, is crucial in these patients.

In our study, we employed a meta-analytic approach to assess the benefits of HFNC over NIV in post-CHS pediatric CHD patients. Key outcome measures, such as reintubation rate, ICU stay length, mechanical ventilation duration, and PaO_2_ and PaCO_2_levels 24 h post oxygen therapy, were evaluated. Our data indicated a statistically significant advantage for HFNC in terms of reduced reintubation rates and shorter ICU stays in comparison to NIV. Jayashankar JP. found in his research that infants in the HFNC group had a significantly lower incidence of interface-related pressure ulcers [13].

Approximately 6–9% of pediatric CHS patients experience postoperative reintubation [17]. The superiority of HFNC in this regard, as observed in our study, could be attributed to its distinctive physiological effects: ① HFNC delivers humidified gas at near body temperature, with a consistent oxygen fraction ranging between 0.21–1.0 and a peak flow rate of 60L/min [18]; ② Its airflow, which often surpasses the patient's peak inspiratory flow rate, minimizes inspiratory resistance, thus reducing the respiratory effort [19, 20], and consequently, the body’s oxygen consumption [21]; ③ The high-flow regime effectively flushes the upper airway's anatomical dead space, facilitating CO_2_elimination and enhancing ventilation [22]; ④ The therapy generates a mild, consistent positive airway pressure that can re-expand collapsed alveoli, thus improving oxygenation by augmenting end-expiratory lung volume and tidal volume [23]. Our study also highlighted the shorter ICU stays with HFNC, though some investigations, like the one by Alok et al., [11] didn't echo this finding. This discrepancy can be attributed to various confounding factors, including patient health status, concurrent complications, and the gravity of primary conditions.

Notably, extended extracorporeal circulation post-CHS poses a significant extubation failure risk, stemming from an augmented inflammatory response, heightened edema, compromised respiratory function, and acute lung injuries, all of which decrease successful extubation likelihood. Prolonged mechanical ventilation, too, is associated with increased extubation failure [24]. In our research, neither extracorporeal circulation duration nor mechanical ventilation duration exhibited any statistical difference between HFNC and NIV groups. This suggests that the evident benefit of HFNC in reducing reintubation rates wasn't confounded by these variables. Some individual studies [11, 12] posited that HFNC enhances oxygen therapy efficiency. However, our meta-analysis didn't discern a significant impact on 24 h PO_2_ and PCO_2_ levels post oxygen therapy, possibly due to the limited observation timeframe or the confined data from included studies. This necessitates more comprehensive RCTs for a definitive conclusion.

Our study had inherent limitations. One of the most important limitations of this study is the inability to differentiate between patient cohorts in terms of disease severity. Although the severity of disease in the subgroups was not specified in the included studies, it is not possible to tell whether the choice of HFNC versus NIV is directly related to the severity of disease. However, in one of the RCT studies, grouping was done on a randomized basis (using opaque envelopes). In the remaining four cohort studies, there was a clear indication that the choice of the mode was based primarily on the discretion of the attending pediatric cardiac intensivist evaluating the infant at the time of extubation. Secondly, The dearth of available RCTs made conclusions tentative. While the unique patient population, blinded to their oxygen therapy type, could signify participant blinding, the inherent specifics of the study methodologies (distinct oxygen therapies) made investigator blinding challenging. This might have compromised the overall quality of our included studies. Additionally, given the limited available data, subsequent research should consider subgroup analysis based on surgery type, patient age, gender, body mass index, extubation time post-surgery, and specific HFNC and NIV device settings.

## Conclusion

Using HFNC in pediatric patients following CHS after extubation decreases the reintubation rate and reduces the length of stay in the CICU compared to NIV. However, it doesn't show a significant improvement in mechanical ventilation duration or oxygenation. Given the limited number of studies in this meta-analysis, the observed heterogeneity, and the constraints of the study design, the conclusions offer limited clinical guidance. More robust RCTs are essential for further validation.

## Data Availability

The data that support the findings of this study are available from the corresponding author of Literature included. But restrictions apply to the availability of these data, which were used under license for the current study, and so are not publicly available.
